# International external quality control assessment for the serological diagnosis of dengue infections

**DOI:** 10.1186/s12879-015-0877-0

**Published:** 2015-04-01

**Authors:** Cristina Domingo, María Joao Alves, Fernando de Ory, Anette Teichmann, Herbert Schmitz, Rolf Müller, Matthias Niedrig

**Affiliations:** Robert Koch Institute, Nordufer 20, 13353 Berlin, Germany; National Institute of Health Dr. Ricardo Jorge, Av. Liberdade 5, 2965 575 Águas de Moura, Portugal; Instituto de Salud Carlos III, Carretera de Majadahonda a Pozuelo, Km 2, 28220 Majadahonda, Madrid Spain; Bernhard Nocht Institute for Tropical Medicine, Bernhard-Nocht-Str. 74, 20359 Hamburg, Germany; Biomatrica Inc., 5627 Oberlin Drive, Suite 120, 92121 San Diego, CA USA

**Keywords:** Dengue, Serology, External quality assessment, Dengue IgM, Dengue IgG

## Abstract

**Background:**

Dengue is endemic to the tropics and subtropics, and the most frequent of arthropod-borne viral diseases. Reliable diagnosis of dengue infection is important not only in clinical care but also in disease surveillance, the control of outbreaks, and the development of new vaccines. The diagnosis of dengue infection is usually established by a variety of commercial or in-house serological protocols. The European Network for the Diagnostics of Imported Viral Diseases (ENIVD) recognized the need to survey the accuracy of dengue serological diagnostics in current use, and organized an external quality assurance (EQA) study of dengue serological practice in diagnostic laboratories.

**Methods:**

A 15-sample panel, consisting of sera reactive against dengue plus specificity and negative controls, was sent to 48 laboratories for serological testing. The results returned by the participating laboratories were anonymized, scored, and subjected to comparison and statistical analysis.

**Results:**

Ten laboratories rated all samples correctly with regard to IgM, and only three achieved the full score for IgG detection. The main handicaps in assay performance were suboptimal sensitivity of in-house IgM detection protocols by comparison with better-performing commercial ELISA tests, and the presence of IgG cross-reactivity with heterologous flaviviruses. Differences of detail in the methodology of dengue IgG antibody detection appear to underlie the disparities in accuracy observed between laboratories.

**Conclusion:**

This EQA study demonstrates that there is room for many laboratories to improve sensitivity in the detection of anti-dengue virus IgM antibodies, against the benchmark set by commercial antibody capture ELISA tests. The EQA shows also that cross-reactivity is a continuing issue, and IgG detection protocols must be optimized to increase their specificity.

**Electronic supplementary material:**

The online version of this article (doi:10.1186/s12879-015-0877-0) contains supplementary material, which is available to authorized users.

## Background

Dengue viruses (DENV; family *Flaviviridae*, genus *Flavivirus*) are transmitted by *Aedes sp.* mosquitoes and comprise four genetically and antigenically distinct serotypes (DENV1—4). Infection with one DENV serotype leads to lifelong protection against a homologous challenge, but only brief cross-protection against heterologous infection [1].

Dengue is one of the most widespread arboviruses. Nearly 2.5 billion adults and children are at risk of dengue infection in the tropics and subtropics, together with 120 million travellers to these regions every year [2]. Children are at a greater risk of life-threatening manifestations of infection [3]. According to World Health Organization (WHO) estimates, 100 million people are infected with DENV annually, and 500,000 develop the more severe dengue haemorrhagic fever (DHF). The incidence of dengue, however, is probably underreported, as endemicity areas include countries where notification is lax and laboratory diagnosis not always available [3].

In Europe, dengue is largely an imported disease, with hundreds of cases every year among European travellers returning from the tropics [4]. Travellers are also potential carriers of the more virulent dengue strains into endemic areas with milder resident strains, but also into non-endemic areas where the mosquito vector is present [5]. Therefore, the recent introduction of *Aedes albopictus* to Europe increases the risk of sustained transmission of the disease within Europe [6,7]. The epidemiology of dengue in Europe has deteriorated over the last few years. Sporadic cases of autochthonous dengue have been reported recently from France [8,9] and Croatia [10,11]. In 2012, Europe experienced the first large, autochthonous dengue outbreak since an outbreak in Greece in the 1920s: a total of 2,103 probable and confirmed cases were reported from the island of Madeira, Portugal [5,12], along with 78 cases imported into other European countries [12]. Regardless of the imported or autochthonous origin of the infection, timely and accurate diagnosis of dengue is crucial to rule out differential diagnoses and guide clinical care, but also in epidemiological surveillance, outbreak intervention, and vaccine development [3,13].

The laboratory diagnosis of dengue relies on tests for DENV infection markers in patient serum. Virus isolation and the detection of viral antigens or genomic RNA can be used for diagnostic purposes during the early phase of illness. At the end of the acute phase of infection, beyond 5 to 6 days after onset, a serological assay for anti-DENV antibodies is the method of choice. Different patterns in the antibody response are observed depending on the primary or secondary nature of dengue infection [13,14]. In primary infections, the IgM antibody response can be measured after the decline of viraemia, between days 3 to 5 after the onset of infection, and persists for approximately six months. In secondary infections, the duration and magnitude of the IgM response are reduced. The IgG antibody response, which in primary infection develops a few days after the onset of the IgM antibody response, may persist for many years. In secondary infections, the IgG response is fast, occurring 2–3 days after illness onset, and of greater magnitude than that in primary infections. Serological tests are widely used for dengue diagnosis because of their convenience and their reduced cost compared to molecular assays. Cross-reactivity with other circulating flaviviruses is the major obstacle in the serological diagnosis of dengue infections, but false positives have also been observed in sera from patients with malaria or leptospirosis [15].

The European Network for Imported Viral Diseases (ENIVD) is a laboratory surveillance network for imported, rare and emerging viral diseases of serious threat to public health. ENIVD is concerned with the development of laboratory diagnostic capability, quality control, standardization of laboratory procedures, and laboratory staff training (http://www.enivd.de) [2]. ENIVD is currently composed by 58 qualified or national reference laboratories (including microbiologists, clinicians, medical scientists, biologists, epidemiologists, chemists, and veterinarians) based at public health institutes and universities in 39 countries, including 27 European Union member states, Norway, Turkey, Bosnia and Herzegovina, Croatia, Kosovo, Serbia, the former Yugoslav Republic of Macedonia, Albania, Switzerland, and Russia. These laboratories support the surveillance and detection of emerging and vector-borne pathogens for the public health institutions in their respective countries [16]. ENIVD regularly organizes external quality assessment (EQA) activities to assess the ability of participating laboratories to correctly detect, identify, characterize and diagnose specific pathogens. We report here on the second ENIVD dengue serology EQA that surveyed the serological diagnostic capacity for dengue infections, and the performance of laboratories using different in-house or commercial assays. This EQA aimed to identify possible weaknesses in the practice of serological tests that could compromise the diagnosis or surveillance of dengue infections.

## Methods

### Participants

Fifty-nine institutions involved in laboratory diagnostics of DENV infection were invited to participate in this study. Invitees were members of the ENIVD, national or regional dengue reference laboratories, and dengue tests manufacturers. The study was announced as an EQA study on DENV serological diagnostic proficiency, which included certifying and publishing the results in a comparative and anonymous manner. Forty-eight laboratories (response rate: 81.3%) took part to the EQA under the coordination of ENIVD: 13 laboratories (27%) from endemic countries, and 35 from non-endemic areas, namely 33 European countries, Iran, and Israel (Additional file 1).

### Specimen preparation and dispatch

**Figure 1:**
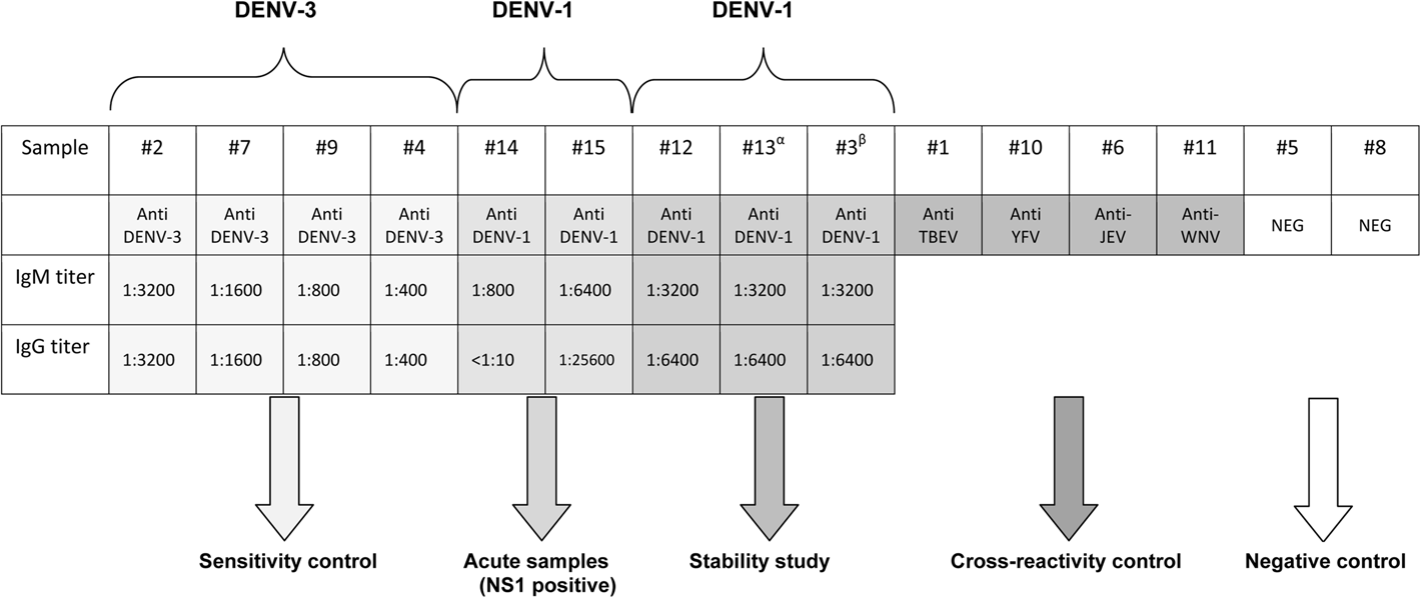
**Dengue serology external quality assurance sample panel composition.** NEG: negative; DENV; dengue virus; TBEV: Tick borne encephalitis virus; YFV: yellow fever virus; JEV: Japanese encephalitis virus; WNV: West Nile virus. α: SM162 reagent included; β: Formulation-C reagent included.

A 15-sample panel (Figure 1) was assembled for distribution to the participating laboratories. Four samples (#2, #7, #9, and #4) represented a sensitivity control consisting of serial two-fold dilutions of a recent dengue infection serum sample from Costa Rica, known to contain both anti-DENV IgM and IgG. Samples #14 (IgM+/IgG±/NS1+) and #15 (IgM+/IgG+/NS1+) were obtained from acutely ill patients during the aforementioned DENV-1 outbreak in Madeira, regarded as the most recent European dengue outbreak. Three further samples examined possible variation in the stability of target antibodies; for this, the same serum sample was formulated by three different procedures: one aliquot was processed by lyophilization as the reference method (#12), another aliquot was lyophilized with a preserving agent (Formulation-C, Biomatrica, CA, USA) that increases the stability of proteins at room temperature (#3), and the third aliquot was simply mixed with a specific protein stabilizer (SM162, from Biomatrica) intended to obviate further processing (#13). In addition, the panel included 4 specificity controls (#1, #10, #6, and #11) in the form of IgM+, IgG+ sera from patients infected by non-dengue flaviviruses (tick-borne, yellow fever, Japanese encephalitis and West Nile virus respectively). Lastly, 2 samples of flavivirus-negative human plasma (#5 and #8) were included as true negative controls.

The non-dengue, flavivirus-positive sera used as specificity controls in the panel were from the Robert Koch Institute collection and their use was approved by the Charité medical ethics committee (EA1/068/10). The positive dengue sera in EQA samples #14 and #15 were provided by author MJA and belong to the reference collection of the Center for the Study of Vectors and Infectious Diseases (CEVDI, Águas de Moura, Portugal). The sera were collected and use in accordance with ethical regulations from the National Institute of Health, Portugal, in a codified database preserving the confidentiality of personal data. The sera purchased from Sera Care Life Sciences (Milford, MA, USA) to prepare the rest of dengue positive samples in the panel (samples #2, #7, #9, #4. #12, #13, and#3) were obtained from volunteers at FDA-regulated donation centers, with strict adherence to federal HIPAA (Health Insurance Portability and Accountability Act) regulations. The study was conducted according to the principles of the Declaration of Helsinki.

The samples tested positive by in-house immunofluorescence assays in the respective laboratories. Sample validation was carried out at the Robert Koch Institute, as follows. The samples were screened by a commercial immunofluorescence assay (Flavivirus IFA Mosaik, Euroimmun AG, Lübeck, Germany). Those samples processed by freeze-drying (all except #13, which was mixed with a stabilizing agent and left in liquid form) were subjected to commercial immunofluorescence assays (Flavivirus IFA Mosaik and Dengue (1–4) IFA Mosaik, both from Euroimmun AG), and quantitatively titrated by ELISA (IgM capture/indirect IgG ELISA, Panbio, Brisbane, Australia). Lastly, qualitative results were validated externally by the National Institute of Health in Águas de Moura, Portugal, and the samples were serotyped at the Bernhard-Nocht Institute in Hamburg, Germany, using an in-house ELISA assay. The sample panel was then shipped to each participating laboratory at room temperature by regular mail, together with directions to reconstitute the samples by adding 100 μl of distilled water, and a reporting chart template for returning the results and relevant methodological information.

### Evaluation of the results

The results from each laboratory were anonymized for data processing under a numerical identification code, with the suffix a, b, or c appended to identify a set among multiple sets sent by one laboratory based on different methods. The results were scored with regard to sensitivity and specificity. One point was given for each correct dengue-positive or dengue-negative assignment. False-negative results received no points, whereas false positive results were penalized with a negative point. Equivocal or borderline results were considered as positive. IgM and IgG results were considered separately. Sample #14 was not scored for IgG as it was known to contain extremely low IgG levels. The complete panel of results was sent to the participants in an anonymous manner so that they could only identify the results from their own laboratory.

### Statistical analysis

The data collected were tabulated in a Microsoft Excel worksheet (Microsoft Corp., Bellingham, WA, USA) and analyzed using the SPSS 14.0 software for Windows (SPSS Inc., Chicago, IL, USA). Evaluation of the analytical sensitivity (the ability to detect true positives) involved the serum samples positive for DENV: #2, #7, #9, #4, #14, #15, #12, #13, and #3. The evaluation of specificity (the ability to correctly assign DENV-negative samples) involved samples #1, #10, #6, #11, #5, and #8. The data on categorized variables, such as scores and the proportion of correct results, were analyzed by Fisher’s exact test, two-tailed. Results were considered statistically significant at the p < 0.05 level.

## Results

The 48 participating laboratories (see Additional file 1) returned a total of 55 sets of IgM results, including three double and two triple sets from laboratories using more than one method in parallel (2a and 2b; 6a, 6b and 6c; 7a, 7b and 7c; 32a and 32b; 46a and 46b), and 47 sets of IgG results, including three double and one triple sets (2a and 2b; 6a and 6b; 7a, 7b and 7c; 32a and 32b), as listed in Additional file 2 and Additional file 3. Forty-three laboratories (89.6%) tested for both IgM and IgG antibodies, and only 6 laboratories (12.5%), mainly from endemic areas, tested for IgM alone in their routine diagnosis (laboratories 21, 38, 42, 44, 47, and 48) (Additional file 1).

A variety of methods were used by participating laboratories to screen the sera: ELISA (IgM, n = 42 [76.4%]; IgG, n = 33 [70.2%]), indirect immunofluorescence (IIF; IgM, n = 11 [20%]; IgG, n = 13 [27.6%]), and rapid immunochromatographic tests (IgM, n = 2 [3.6%]; IgG, n = 1 [2.1%]). In-house assays contributed 1 in 7 IgM data sets (ELISA, n = 5 [11.9%]; IIF, n = 3 [27.3%]), and 1 in 6 IgG data sets (ELISA n = 4 [12.1%]; IIF n = 4 [30.8%]). Among the data involving commercial tests, the Panbio ELISA assays were those used most frequently (IgM: 11 out of 47 data sets [23.4%]; IgG: 11 out of 39 data sets [28.2%]), followed by the Novatec ELISA assays (IgM: 8 out of 47 data sets [17%]; IgG: 6 out of 39 [15.4%]). No laboratories reported the use of haemagglutination inhibition or immunoblot tests.

Dengue NS1 antigen detection, which was not part of the EQA specification, was performed by only four laboratories but we can not rule out wider application of this assay in the routine diagnosis of acute cases.

The mean score overall for IgM detection was 11.5, with 78.5% correct positive or negative sample assignments on average (Additional file 2), but performance varied among laboratories depending on the assay used: ELISA (mean score of 12 and 81.3% average correct assignments), IIF (9.6 and 69.7%) or rapid immunochromatographic tests (10 and 67%). The mean score achieved by laboratories using commercial assays was 12.1 (returning 82% correct results on average), while the mean score for those using in-house techniques was 8.8 (64.4% average correct results). Among IgM assays, statistically significant differences in the proportion of correct results were observed between ELISA and IIF (p < 0.001), and between commercial and in-house assays (p < 0.0001).

As regards the 55 sets of results for IgM antibodies, 10 out of the 11 laboratories using the Panbio Dengue IgM capture assay achieved a full set of 15 correct calls across the sample panel, followed by 5 of the 6 laboratories using the Dengue Virus IgM Capture DxSelect test from Focus Diagnostics with 14 correct calls. Lower scores by the other assays issued mainly from incorrect IgM non-detection. The laboratories using the Novatec Indirect Dengue IgM ELISA failed to detect IgM below a titre of 1:1600. The Euroimmun IIF and ELISA assays displayed lower sensitivity on samples #7, #9, and #4, which may be related to the DENV-3 serotype shared by these samples. The sensitivity of the SD Dengue Duo IgM/NS1 and IgM capture ELISA tests was restricted to samples with a titre above 1:3200 (Additional file 2).

Conclusions can be drawn about the sensitivity and specificity of the different IgM detection methods from same-sample comparisons of the rate of correct calls achieved by each, notably from the comparison between ELISA and immunofluorescence assays. The overall sensitivity for IgM detection was 66.7% (95% confidence interval (CI): 62.3% to 70.8%) with 95.1% specificity (95% CI: 92.2% to 97.2%). Laboratories using commercial ELISA tests showed the highest sensitivity compared with those using commercial IIF and in-house ELISA or IIF assays (Table 1). The commercial IIF assays were of limited sensitivity in detecting the serological immune response against DENV-3, while their sensitivity to anti-DENV-1 IgM antibodies was much better, as was their specificity profile (Table 2). The high sensitivity demonstrated through the use of commercial IgM capture formats clearly stands out by comparison with indirect assays, which were on a level with IIF assays (Table 1).

**Table 1:** **Analytical sensitivity and specificity showed by the different ELISA and IIF assays used by the participant laboratories**

	**IgM sensitvity**	**IgM specificity**	**IgG sensitivity**	**IgG specificity**
Overall*	66.7% (95% CI 62.3%-70.8%)	95.1% (95% CI 92.2%-97.2%)	84.4% (95% CI 80.6%-87.7%)	83% (95% CI 78%-87.1%)
**ELISA assays**	70.4% (95% CI 65.5%-75%)	97.2% (95% CI 94.4%-98.9%)	83.2% (95% CI 78.5%-87.3%)	83.8% (95% CI 78%-88.7%)
ELISA commercial	75.5% (95% CI 70.3%-80.2%)	97% (95% CI 93.7%-98.9%)	82.4% (95% CI 77.3%-86.8%)	84.5% (95% CI 78.2-89.5%)
*Capture assays*	82.7% (95% CI 77%-87.4%)	96% (95% CI 91.5%-98.6%)	59.3% (95% CI 45%-72.4%)	97.2% (95% CI 85.4%-99.5%)
*Indirect assays*	55.6% (95% CI 44%-66.6%)	100% (95% CI 93.3%-100%)	88.9% (95% CI 83.9%-92.9%)	82.6% (95% CI 75.2%-88.5%)
ELISA in house	48.6% (95% CI 35.6%-60.7%)	97.9% (95% CI 88.9%-99.6%)	88.9% (95% CI 73.9%-96.8%)	79.2% (95% CI 57.8%-92.8%)
**IIF assays**	56.6% (95% CI 46.2%-66.5%)	86.4% (95% CI 75.7%-93.6%)	88.9% (95% CI 81.7%-93.9%)	79.5% (95% CI 68.8%-87.8%)
IIF commercial	59.7% (95% CI 47.5%-71.1%)	97.9% (95% CI 88.9%-99.6%)	88.9% (95% CI 79.9%-94.8%)	77.7% (95% CI 64.4%-87.9%)
IIF in house	48.1% (95% CI 28.7%-68%)	55.5% (95% CI 30.8%-78.4%)	88.9% (95% CI 73.9%-96.8%)	83.3% (95% CI 62.6%-95.2%)

*ELISA, IIF, and rapid test included. Due to the small number of data sets, rapid test were excluded from further analyses.

**Table 2:** **Number of correct IgM qualitative results per panel sample and technology type**

					**ELISA**				**IIF**				**Rapid test** ^**d**^	
**Sample code**	**Sample content**	**Sample titer**	**Total data sets**		**In-house** ^**a**^		**Commercial** ^**b**^		**In-house** ^**a**^		**Commercial** ^**c**^			
			**n = 55**		**n = 8**		**n = 34**		**n = 3**		**n = 8**		**n = 2**	
			**n**	**%**	**n**	**%**	**n**	**%**	**n**	**%**	**n**	**%**	**n**	%*
Den#02	DENV-3	1:3200	35	63.6	3	37.5	27	79.4	0	0	5	62.5	0	0
Den#07	DENV-3	1:1600	28	50.9	3	37.5	21	61.8	1	33.3	1	12.5	0	0
Den#09	DENV-3	1:800	21	38.2	1	12.5	16	47	2	66.6	2	6.25	0	0
Den#04	DENV-3	1:400	12	21.8	0	0	11	32.3	1	33.3	0	0	0	0
Den#12	DENV-1	1:3200	50	90.9	6	75	32	94.1	1	33.3	7	87.5	2	100
Den#14	DENV-1	1:800	29	52.7	1	12.5	22	64.7	2	66.6	5	62.5	0	0
Den#15	DENV-1	1:6400	54	98.1	7	87.5	34	100	3	100	8	100	2	100
Den#13	DENV-1	1:3200	51	92.7	7	87.5	34	100	1	33.3	7	87.5	2	100
DEN#03	DENV-1	1:3200	53	96.3	7	87.5	34	100	2	66.6	8	100	2	100
DEN#01	TBEV		53	96.3	7	87.5	33	97	3	100	8	100	2	100
DEN#10	YFV		51	92.7	8	100	33	97	1	33.3	7	87.5	2	100
DEN#06	JEV		52	94.5	8	100	33	97	2	66.6	8	100	2	100
DEN#11	WNV		52	94.5	8	100	33	97	1	33.3	8	100	2	100
DEN#05	NEG		53	96.3	8	100	34	100	1	33.3	8	100	2	100
DEN#08	NEG		53	96.3	8	100	32	94.1	2	66.6	8	100	2	100

NEG: negative; DENV; dengue virus; TBEV: Tick borne encephalitis virus; YFV: yellow fever virus; JEV: Japanese encephalitis virus; WNV: West Nile virus, IIF: indirect immunofluorescence.

*not statistically representative.

a: Details not presented; b: PanBio IgM capture ELISA (n = 10), NovaTec Dengue virus IgM-ELISA (n = 8), FOCUS Dengue virus IgM capture DxSelect (n = 6), Euroimmun Anti-Dengue Virus ELISA (IgM) (n = 3), InBios DENV Detect IgM capture ELISA (n = 2), Standard Diagnostics (SD) Dengue IgM capture ELISA (n = 2), Vircell IgM ELISA (n = 2), Dengue virus IgM ELISA IBL International (n = 1); c: Euroimmun DEN1-4 IFA Mosaik (n = 6), Euroimm Flavivirus IFA Mosaik (n = 2); d: Standard diagnostics (SD) dengue Duo IgM/NS1 (n = 2).

Concerning the specificity of the different assays, no major problems were noted in the results of IgM detection returned by the majority of laboratories. Nonetheless, six sets of results presented cross-reactivity on non-dengue flaviviral infection samples, and three laboratories made false-positive calls on the negative control samples, #5 and #8 (Additional file 2). Two laboratories in the EQA reported performing a confirmatory background substraction assay following isolated IgM detection [14] in sample #14. Results from commercial and in-house ELISA tests were of comparable specificity, and no differences in specificity were observed between IgM capture and indirect assays. In this regard, however, those laboratories using in-house IIF assays were at a clear disadvantage compared to those reporting the use of commercial IIF tests, which showed here a specificity profile similar to that of the ELISA assays (Table 1 and Table 2).

Regarding anti-dengue IgG detection, only three laboratories achieved a full set of correct assignments across the EQA sample panel. Performance was not related to a specific method, and optimal results were obtained with different assays (Additional file 3); overall sensitivity was 84.4% (95% CI: 80.6%-87.7%), and specificity was 83% (95% CI: 78%-87.1%) (Table 1). The mean score for IgG detection was 11.5 with 89.3% correct results on average, similar to the results from ELISA (11.5 and 89.1%) and IIF (11.5 and 90.2%) assays. The mean score for laboratories using commercial assays was 11.4 (89% correct results), and 11.7 (91.1% correct results) for laboratories using in-house tests. No statistically significant differences were found between the correct call rates achieved with the different tests (p > 0.05) with sensitivities ranging from 82.4% to 88.9% (Table 1).

No major sensitivity issues were observed across the anti-DENV IgG-positive samples in the test panel, but 34 out of 47 sets of results (72.3%) included a cross-reactive, false-positive assignment of the serum from a WNV-infected patient (sample #11). Low specificity is mentioned as a potential complication by commercial manufacturers in their instructions, and occurred here in laboratories applying different assays. No statistically significant differences in the number of false positives were observed between laboratories using commercial versus in-house protocols, or ELISA versus IIF tests. A review of the proportion of correct results for each serum sample revealed no differences between these assay categories with regard to specificity (Table 3).

**Table 3:** **Number of correct IgG qualitative results per panel sample and technology type**

**Sample code**	**Sample content**	**Sample titer**			**ELISA**				**IIF**				**Rapid test** ^**d**^	
			**Total data sets**		**In-house** ^**a**^		**Commercial** ^**b**^		**In-house** ^**a**^		**Commercial** ^**c**^			
			**n = 47**		**n = 4**		**n = 29**		**n = 4**		**n = 9**		**n = 1**	
			**n**	**%**	**n**	**%**	**n**	**%**	**n**	**%**	**n**	**%**	**n**	%*
Den#02	DENV-3	1:3200	47	100	4	100	29	100	4	100	9	100	1	100
Den#07	DENV-3	1:1600	43	91.5	4	100	26	89.6	4	100	8	88.8	1	100
Den#09	DENV-3	1:800	41	87.2	4	100	24	82.7	4	100	9	100	0	0
Den#04	DENV-3	1:400	38	80.8	4	100	22	75.9	4	100	8	88.8	0	0
Den#12	DENV-1	1:6400	47	100	4	100	28	96.5	4	100	9	100	1	100
Den#14	DENV-1	<1:10	5	10.6	0	0	3	10.3	0	0	2	22.2	0	0
Den#15	DENV-1	1:25600	47	100	4	100	29	100	4	100	9	100	1	100
Den#13	DENV-1	1:6400	46	97.9	4	100	28	96.5	4	100	9	100	1	100
DEN#03	DENV-1	1:6400	45	95.7	4	100	27	93.1	4	100	9	100	1	100
DEN#01	TBEV		45	95.7	4	100	29	100	4	100	7	77.7	1	100
DEN#10	YFV		45	95.7	4	100	28	96.5	4	100	8	88.8	1	100
DEN#06	JEV		41	87.2	4	100	25	86.2	4	100	7	77.7	1	100
DEN#11	WNV		13	27.6	1	25	9	31	0	0	2	22.2	1	100
DEN#05	DENV neg.		45	95.7	3	75	28	96.5	4	100	9	100	1	100
DEN#08	DENV neg.		45	95.7	3	75	28	96.5	4	100	9	100	1	100

NEG: negative; DENV; dengue virus; TBEV: Tick borne encephalitis virus; YFV: yellow fever virus; JEV: Japanese encephalitis virus; WNV: West Nile virus, IIF: indirect immunofluorescence;*not statistically representative.

a: Details not presented; b: PanBio indirect IgG ELISA (n = 11), NovaTec Dengue virus IgG-ELISA (n = 6), FOCUS Dengue Virus IgG DxSelect (n = 5), Euroimmun Anti-Dengue Virus ELISA (IgG) (n = 2), InBios Dengue Detect IgG Capture ELISA (n = 1), Standard Diagnostics (SD) Dengue IgG capture ELISA (n = 2), Dengue Virus IgG ELISA IBL International (n = 1); c: Euroimmun IFA Dengue 1–4 Mosaik (n = 7), Euroimmun IFA Flavivirus Mosaik (n = 2) d: Standard diagnostics (SD) dengue IgM/IgG (n = 1).

In the results for the cross-reactivity control samples, incorrect IgG-positive calls were made by some, but not all, laboratories using the dengue virus IgG DX Select assay from Focus Diagnostics or the Novatec dengue virus IgG ELISA. In addition, only some of the laboratories using the Euroimmun Flavivirus Mosaik assay correctly identified the presence of antibodies against other (non-dengue) flaviviruses. Overall, this suggests that the specificity profile does not depend exclusively on assay design but also on the actual details of laboratory procedures. The laboratories using the assays from Standard Diagnostics (Dengue IgG capture ELISA and Bioline dengue IgM/IgG) were free from cross-reactivity in their results, probably at the cost of lower sensitivity. Likewise, three laboratories (participants #35, 36, and 45) using the Panbio Dengue IgG capture assay avoided a false-positive call on sample #11 containing anti-WNV antibodies. These laboratories reported the use of a higher cut-off value for IgG levels in secondary infections, so that they also experienced a loss in sensitivity (Additional file 3). Laboratories using commercial capture assays for dengue IgG detection clearly achieved a higher degree of specificity than those using indirect formats, but this was associated with much lower sensitivity (Table 1).

## Discussion

We have reviewed here the results from the second DENV serological diagnosis EQA, carried out under the auspices of the ENIVD network. An external quality assessment of laboratory diagnostics such as this one is a process to survey the sensitivity and specificity of current methods for detecting viral infection, identify weaknesses in the proficiency of individual laboratories, and provide participating centres with advice and assistance. There is increasing recognition among the dengue community of the need for quality assessments, and the present study brought together a higher number of laboratories, 48, than in previous occasions. Results were gathered from laboratories in countries with different epidemiological situations (endemic versus non-endemic), which had an impact on the diversity of the methods reported and the complexity of the data collected. Laboratories from endemic areas, in particular, may provide information on the performance of methods favoured locally (i.e. assays from Standard Diagnostics), the use of different diagnosis algorithms (diagnosis of primary versus secondary infections), or the preparedness for surveillance of countries where this competence is most needed.

The design of the EQA enabled participants to test well-characterized samples of different origin and measure their own performance against that of other laboratories. In this way, they may identify shortcomings in their protocols which otherwise would remain undetected. They also gained access to valuable reference material that they may use after the EQA for the improvement or development of their assays. The comprehensive panel of samples distributed to the participating laboratories included sera of patients from endemic areas, infected with either DENV-1 or DENV-3, and also two DENV-1 sera from the most recent outbreak of autochthonous dengue in Europe, which affected the island of Madeira. The inclusion of sera from non-dengue flaviviral infections made the EQA data particularly valuable, as it is well-recognized that cross-reactivity is the major barrier to specific serological diagnostics for a target flavivirus. This may constitute a serious problem for surveillance in countries where more than one flavivirus circulates [3].

This EQA focused at the outset on the diagnostic accuracy of individual laboratories, and the body of data was ultimately large enough to provide indications on the performance of the different assays in use. Superior sensitivity was attained by laboratories using capture ELISA tests for the detection of IgM, specifically those from Panbio and Focus. Laboratories performing other assays, like the Euroimmun Flavivirus and Dengue Mosaik assays, varied in their results depending mainly on the laboratory. In this regard, it must be noted that immunofluorescence-based assays can be very useful tools but their results depend very much on the expertise of the operator and the quality of the microscopy. This was particularly evident in the panel of IgG results where, among laboratories using the same immunofluorescence-based assay, only some were able to discriminate between dengue and an infection by another flavivirus.

Since the finding that sera from patients with coincident diseases may give false-positive IgM, some assay manufacturers (e.g. Focus) recommend the use of a confirmatory assay in their instructions. Only two laboratories in the EQA reported performing a confirmatory background substraction assay following isolated IgM detection in a sample. Although many laboratories are aware of the potential for false-positives in their routine diagnostics and of the advantages of a confirmatory background substraction assay, they opt against this additional test as it would double the cost per sample.

We observed no significant differences in IgG assay performance between commercial and in house protocols, or between different test formats (Table 1). There was a marked difference, by contrast, between commercial and in-house tests for IgM. Indeed, in-house IgM ELISA tests are generally of lower sensitivity compared to commercial methods, where sensitivity is greatly improved through the antibody capture approach. In parallel, commercial IIF assays for IgM detection were found to detect DENV-1 infection with higher sensitivity than DENV-3 (Table 2). This may explain the lower score average and percentage of correct results in IgM detection by IIF compared with ELISA assays.

The high rate of IgG false positives reported by the participants for the sample containing anti-WNV antibodies is striking. This false reactivity has been recognized by the manufacturers of commercial assays, but the question remains on how to address it in countries with WNV circulation. Higher specificity was attained by some laboratories using assays aimed at detecting secondary infections, namely the Standard Diagnostics or the Panbio Dengue IgG capture assay, though at the cost of lower sensitivity. As these laboratories experienced also less cross-reactivity with tick-borne encephalitis, yellow fever, or Japanese encephalitis, this alternative approach deserves consideration.

Lastly, a small number of laboratories made false-positive calls on the negative control samples (Additional file 2 and Additional file 3), which is suggestive of deficiencies in their operating procedures or poor standardization of in-house protocols.

A critical point in the design of an international EQA exercise is the shipment of samples. ENIVD freeze-dried sample panels are always sent by regular mail at room temperature. Sample quality may arguably be affected by both the preparation process and by delivery time to the laboratory: in some instances, up to several weeks under tropical conditions. However, experience from this and previous EQAs shows that the impact of temperature and delivery time on sample quality is very limited or non-existent. Only in a few cases have changes in the physical characteristics of the material been reported (one laboratory [#33] in this EQA reported difficulties in reconstituting the samples but its results were not affected). Similarly, the results obtained by the laboratories show that the quality of samples was not a limitation in the EQA. Nevertheless, we are interested in improving the quality of the ENIVD EQA panels and different sample stabilizers are being tested for that purpose. Here, a reference sample was prepared by freeze-drying, and two additional aliquots of the same sample were respectively prepared using two commercial stabilizers. Assay performance was consistent across the three related samples. A serum with a high antibody titre was selected for this stability test; in future EQAs, samples with a titre closer to the limit of detection may be included for a more stringent assessment of the effect of stabilizing reagents on assay sensitivity.

A previous EQA on dengue serology was performed by ENIVD in 2002, and eleven laboratories participated in both this and the present EQA [17]. Direct performance comparisons between the two EQAs are not possible, because the 2002 panel included no sera from non-dengue flaviviral infections, nor was quantitative titration used in the initial characterization of the samples.

## Conclusions

From the results of this EQA, we conclude that the quality of dengue IgM-based serological diagnosis depends on the type of protocol used by the laboratories, with those using commercial antibody capture ELISA formats significantly outperforming the others. We hope that the results we describe will help improve the sensitivity standards for IgM detection among the dengue community. The performance of IgG detection seemed to be linked mostly to particulars of a technique as practiced by individual laboratories, and cross-reactivity remains a serious issue. IgG capture ELISA formats seem to provide better specificity, but the trade-off observed between specificity and sensitivity has to be considered.

## Additional files

Additional file 1:
**Participating laboratories in the Dengue Serology External Quality Assurance activity.**
Additional file 2:
**Results on Dengue virus IgM serology per laboratory.**
Additional file 3:
**Results on Dengue virus IgG serology per laboratory.**
