# Supplementary material for: International external quality control assessment for the serological diagnosis of dengue infections
Source: BMC Infect Dis. 2015 Apr 1;15:167. doi: 10.1186/s12879-015-0877-0 (PMC4392463; doi:10.1186/s12879-015-0877-0)
Supplement: Additional file 1: — Participating laboratories in the Dengue Serology External Quality Assurance activity. [file 12879_2015_877_MOESM1_ESM.docx]

Additional file 1. Participating laboratories in the Dengue Serology External Quality Assurance activity

| Institute | IgM results | IgG results |
| --- | --- | --- |
| Virology Department, School of Medicine, Vienna University, Austria | X | X |
| Central Laboratory of Clinical Biology, Institute of Tropical Medicine, Belgium | X | X |
| National Reference Laboratory for Arboviruses, Dpt. of Virology, National Institute of Public Health, Czech Republic | X | X |
| Statens Serum Institut, Denmark | X | X |
| National Reference Center for Arboviruses, France | X | X |
| Emerging Viruses Unit, School of Medicine-La Timone Hospital, France | X | X |
| Laboratory for Virology and Molecular Diagnostics, Institute of Public Health of R. Macedonia; FYROM | X | **·** |
| Bernhard Nocht Institut für Tropenmedizin, Germany | X | X |
| Institute of Microbiology, Federal Armed Forces Central area of diagnostics, Germany | X | X |
| EUROIMMUN AG, Germany | X | X |
| NovaTec Immundiagnostica GmbH | X | X |
| Department of Microbiology, School of Medicine, Aristotle University, Greece | X | X |
| Hellenic Pasteur Institut, Greece | X | X |
| Dept. of Viral Diagnostics, National Center for Epidemiology, Hungary | X | X |
| National Virus Reference Laboratory, University College Dublin, Ireland | X | X |
| Molecular Biology Section, Army Medical and Veterinary Research Center, Italy | X | X |
| Molecular Virology Unit, Virology and Microbiology IRCCS, Fondatione Policlinico San Matteo, Italy | X | X |
| Laboratory of Virology, National Institute for Infectious Diseases "L Spallanzani", Italy | X | X |
| School of Medicine, Padova University, Italy | X | X |
| Laboratory of Microbiology and Virology, Amedeo Hospital, Italy | X | X |
| Virology Dept., Infectology Center of Latvia, Latvia | X | X |
| National Public Health Surveillance Laboratory, Lithuania | X | X |
| Div. of Infectious Disease Control, Norwegian Institute of Public Health, Norway | X | X |
| Center for Vectors and Infectious Diseases, National Institute of Health, Portugal | X | X |
| Clinical Pathology Laboratory, Dr. Nélio Mendonça Hospital, Portugal | X | X |
| Faculty of Medicine, University of Ljubljana, Slovenia | X | X |
| National Center for Microbiology, Institute of Health Carlos III (ISCIII), Spain | X | X |
| Swedish Institute for Infectious disease control, Sweden | X | X |
| Virology Labor Spiez, Switzerland | X | X |
| Virology Department, Erasmus University Medical Center, The Netherlands | X | X |
| National Institute of Public Health and the Environment (RIVM), The Netherlands | X | X |
| Health Protection Agency (HPA), UK | X | X |
| School of Clinical Medicine and Research, University of West Indies, Barbados | X | **·** |
| Grupo Virología, National Institute of Health, Colombia | X | X |
| Institute Pasteur of Cameroon, Cameroon | X | X |
| Shanghai Municipal Center for Disease Control Prevention, China | X | X |
| Manipal Centre for Virus Research, Manipal University, India | X | X |
| Pasteur Institute of Iran, Iran | X | X |
| Central Virology Laboratory, Public Health Laboratories-Sheba Medical Center, Israel | X | X |
| Institute Pasteur Madagascar, Madagascar | X | X |
| King Fahd Medical Research Center, King Abdulaziz University Hospital, Saudi Arabia | X | X |
| Institute Pasteur de Dakar, Senegal | X | X |
| Duke-NUS Graduate Medical School, Singapore | X | X |
| Center for Emerging Zoonotic Diseases, (NICD) , South Africa | X | X |
| Uganda Virus Research Institute, Uganda | X | **·** |
| Oxford University Clinical Research Unit (HCMC), Vietnam | X | **·** |
| Faculty of Medical Laboratory, Hospital for Tropical Diseases (HTD), Vietnam | X | **·** |

X: sent results; ·: no results available
