# Supplementary material for: International external quality control assessment for the serological diagnosis of dengue infections
Source: BMC Infect Dis. 2015 Apr 1;15:167. doi: 10.1186/s12879-015-0877-0 (PMC4392463; doi:10.1186/s12879-015-0877-0)
Supplement: Additional file 2: — Results on Dengue virus IgM serology per laboratory. [file 12879_2015_877_MOESM2_ESM.doc]

**Additional file 2.** Results on Dengue virus IgM serology per laboratory

|  |  | anti-DENV-3 | anti-DENV-3 | anti-DENV-3 | anti-DENV-3 | anti-DENV-1 | anti-DENV-1 | anti-DENV-1 | anti-DENV-1^α^ | anti-DENV-1^β^ | anti-TBEV | anti-YFV | anti-JEV | anti-WNV | NEG | NEG |  |  |  |
| --- | --- | --- | --- | --- | --- | --- | --- | --- | --- | --- | --- | --- | --- | --- | --- | --- | --- | --- | --- |
|  | Sample n° | #2 | #7 | #9 | #4 | #12 | #14 | #15 | #13 | #3 | #1 | #10 | #6 | #11 | #5 | #8 | % correct |  |  |
|  | Titer | 1:3200 | 1:1600 | 1:800 | 1:400 | 1:3200 | 1:800 | 1:6400 | 1:3200 | 1:3200 |  |  |  |  |  |  | results | Score | Assay |
| Laboratory ID number | 18 | + | + | + | + | + | + | + | + | + | - | - | - | - | - | - | 100 | 15 | A |
|  | 13 | + | + | + | + | + | + | + | + | + | - | - | - | - | - | - | 100 | 15 | A |
|  | 31 | + | + | + | + | + | + | + | + | + | - | - | - | - | - | - | 100 | 15 | A |
|  | 29 | + | + | + | + | + | + | + | + | + | - | - | - | - | - | - | 100 | 15 | A |
|  | 37 | + | + | + | + | + | + | + | + | + | - | - | - | - | - | - | 100 | 15 | A |
|  | 35 | + | + | + | + | + | + | + | + | + | - | - | - | - | - | - | 100 | 15 | A |
|  | 36 | + | + | + | + | + | + | + | + | + | - | - | - | - | - | - | 100 | 15 | A |
|  | 1 | + | + | + | + | + | + | + | + | + | - | - | - | - | - | - | 100 | 15 | A |
|  | 45 | + | + | + | + | + | + | + | + | + | - | - | - | - | - | - | 100 | 15 | A |
|  | 3 | + | + | + | + | + | + | + | + | + | - | - | - | - | - | - | 100 | 15 | A |
|  | 11 | + | + | + | - | + | + | + | + | + | - | - | - | - | - | - | 93 | 14 | B |
|  | 28 | + | + | + | - | + | + | + | + | + | - | - | - | - | - | - | 93 | 14 | B |
|  | 20 | + | + | (+/−) | - | + | + | + | + | + | - | - | - | - | - | - | 93 | 14 | B |
|  | 21 | + | + | (+/−) | - | + | + | + | + | + | - | - | - | - | - | - | 93 | 14 | B |
|  | 4 | + | + | + | - | + | + | + | + | + | - | - | - | - | - | - | 93 | 14 | B |
|  | 14 | + | + | + | - | + | - | + | + | + | - | - | - | - | - | - | 87 | 13 | N |
|  | 47 | + | + | - | - | + | + | + | + | + | - | - | - | - | - | - | 87 | 13 | N |
|  | 24 | (+/−) | (+/−) | - | - | + | + | + | + | + | - | - | - | - | - | - | 87 | 13 | C |
|  | 46a | + | + | - | - | + | (+/−) | + | + | + | - | - | - | - | - | - | 87 | 13 | D |
|  | 32a | + | + | + | - | + | + | + | + | + | - | + | - | - | - | - | 87 | 12 | F |
|  | 6b | + | - | - | - | + | (+/−) | + | + | + | - | - | - | - | - | - | 80 | 12 | D |
|  | 46b | + | + | - | - | + | - | + | + | + | - | - | - | - | - | - | 80 | 12 | E |
|  | 5 | + | + | - | - | + | - | + | + | + | - | - | - | - | - | - | 80 | 12 | D |
|  | 39 | + | + | - | - | + | - | + | + | + | - | - | - | - | - | - | 80 | 12 | A |
|  | 41 | + | + | - | - | + | - | + | + | + | - | - | - | - | - | - | 80 | 12 | M |
|  | 44 | (+/−) | - | - | - | + | (+/−) | + | + | + | - | - | - | - | - | - | 80 | 12 | C |
| Laboratory ID number | 23 | + | + | - | - | + | + | + | + | + | - | - | - | - | - | - | 80 | 12 | G + F |
|  | 25 | + | - | - | - | + | + | + | + | + | - | - | - | - | - | - | 80 | 12 | F |
|  | 12 | + | - | - | - | + | (+/−) | + | + | + | - | - | - | - | - | - | 80 | 12 | G + F |
|  | 6a | (+/−) | - | - | - | + | (+/−) | + | + | + | - | - | - | - | - | - | 80 | 12 | G |
|  | 48 | (+/−) | + | - | - | + | + | + | + | + | - | - | - | - | - | + | 80 | 11 | I |
|  | 9 | + | - | - | - | + | - | + | + | + | - | - | - | - | - | - | 73 | 11 | D |
|  | 40 | + | - | - | - | + | - | + | + | + | - | - | - | - | - | - | 73 | 11 | D |
|  | 19 | - | - | + | - | + | - | + | + | + | - | - | - | - | - | - | 73 | 11 | G |
|  | 30 | - | - | - | - | + | + | + | + | + | - | - | - | - | - | - | 73 | 11 | J |
|  | 10 | + | + | + | + | + | - | + | + | + | - | - | + | + | - | - | 80 | 10 | B |
|  | 6c | - | - | - | - | + | - | + | + | + | - | - | - | - | - | - | 67 | 10 | E |
|  | 32b | - | - | - | - | + | - | + | + | + | - | - | - | - | - | - | 67 | 10 | H |
|  | 15 | - | - | - | - | + | - | + | + | + | - | - | - | - | - | - | 67 | 10 | H |
|  | 16 | - | - | - | - | + | - | + | + | + | - | - | - | - | - | - | 67 | 10 | H |
|  | 26 | - | - | - | - | + | - | + | + | + | - | - | - | - | - | - | 67 | 10 | F |
|  | 49 | (+/−) | - | - | - | - | - | + | + | + | - | - | - | - | - | - | 67 | 10 | K |
|  | 8 | - | - | - | - | + | - | + | + | + | - | - | - | - | - | - | 67 | 10 | O |
|  | 7b | - | - | - | - | + | - | + | + | + | - | - | - | - | - | - | 67 | 10 | N |
|  | 38 | - | - | - | - | + | - | + | + | + | - | - | - | - | - | - | 67 | 10 | M |
|  | 22 | - | - | - | - | + | - | + | + | + | - | - | - | - | - | - | 67 | 10 | L |
|  | 7c | - | - | - | - | + | - | + | + | + | - | - | - | - | - | - | 67 | 10 | L |
|  | 7a | - | - | - | - | + | - | + | + | + | - | - | - | - | - | - | 67 | 10 | J |
|  | 17 | - | - | - | - | + | - | + | - | + | - | - | - | - | - | - | 60 | 9 | F |
|  | 43 | - | - | - | - | + | - | + | - | + | - | - | - | - | - | - | 60 | 9 | N |
|  | 33 | - | - | - | - | - | - | + | + | + | + | - | - | - | - | - | 53 | 7 | N |
|  | 42 | - | - | - | - | - | - | - | + | - | - | - | - | - | - | - | 47 | 7 | N |
|  | 34 | - | - | - | - | - | + | + | + | + | + | + | + | - | - | - | 47 | 4 | I |
|  | 2a | - | + | + | + | + | + | + | - | + | - | + | + | + | + | + | 53 | 3 | O |
|  | 2b | - | - | + | - | - | + | + | - | - | - | + | - | + | + | - | 40 | 3 | O |
|  | Total | 35 | 28 | 21 | 12 | 50 | 29 | 54 | 51 | 53 | 53 | 51 | 52 | 52 | 53 | 53 | Average | Average |  |
|  | % | 63.6 | 50.9 | 38.1 | 21.8 | 90.9 | 52.7 | 98.1 | 92.7 | 96.3 | 96.3 | 92.7 | 94.5 | 94.5 | 96.3 | 96.3 | 78.47 | 11.47 |  |

α: SM162 preservant; β: Formulation-C preservant

(+/−): Equivocal result; Grey: False positive/negative result

NEG: negative; DENV; dengue virus; TBEV: Tick borne encephalitis virus; YFV: yellow fever virus; JEV: Japanese encephalitis virus; WNV: West Nile virus

Assay code: A: PanBio IgM capture ELISA; B: ELISA IgM capture Dx Select Focus Diagnostic; C: DENV Detect IgM capture ELISA InBios; D: EIA Novatec indirect Dengue IgM; E: EIA IgM μ-capture NovaTec; F: Euroimmun flavivirus IFA Mosaik; G: Euroimmune den 1–4 mosaik IFA; H: Euroimmun anti DEN ELISA; I: Vircell IgM capture ELISA; J: SD Dengue IgM capture ELISA; K: IBL Dengue IgM indirect ELISA; L: SD Dengue Duo IgM and NS1; M: IgM in house CDC MAC- ELISA; N: in house MAC-ELISA; O: in house IFA
