# Supplementary material for: International external quality control assessment for the serological diagnosis of dengue infections
Source: BMC Infect Dis. 2015 Apr 1;15:167. doi: 10.1186/s12879-015-0877-0 (PMC4392463; doi:10.1186/s12879-015-0877-0)
Supplement: Additional file 3: — Results on Dengue virus IgG serology per laboratory. [file 12879_2015_877_MOESM3_ESM.doc]

**Additional file 3.** Results on Dengue virus IgG serology per laboratory

|  |  | anti-DENV-3 | anti-DENV-3 | anti-DENV-3 | anti-DENV-1 | anti-DENV-1 | anti-DENV-1 | anti-DENV-1^α^ | anti-DENV-1^β^ | anti-TBEV | anti-YFV | anti-JEV | anti-WNV | NEG | NEG |  |  |  |
| --- | --- | --- | --- | --- | --- | --- | --- | --- | --- | --- | --- | --- | --- | --- | --- | --- | --- | --- |
|  | Sample | #7 | #9 | #4 | #12 | #14 | #15 | #13 | #3 | #1 | #10 | #6 | #11 | #5 | #8 | % correct |  |  |
|  | Titer | 1:3200 | 1:1600 | 1:800 | 1:400 | <1:10 | 1:6400 | 1:6400 | 1:6400 |  |  |  |  |  |  | results | Score | Assay |
| Laboratory ID number | 33 | + | + | (+/−) | + | - | + | + | + | - | - | - | - | - | - | 100 | 14 | L |
|  | 32a | + | + | + | + | - | + | + | + | -* | - | -* | -* | - | - | 100 | 14 | E |
|  | 20 | + | + | + | + | + | + | + | + | - | - | - | - | - | - | 100 | 14 | B |
|  | 19 | + | + | - | + | - | + | + | + | - | - | - | - | - | - | 93 | 13 | E |
|  | 10 | + | + | - | + | + | + | + | + | - | - | - | - | - | - | 93 | 13 | B |
|  | 46a | + | + | + | + | - | + | + | - | - | - | - | - | - | - | 93 | 13 | D |
|  | 34 | + | + | + | + | - | + | + | + | - | - | - | (+/−) | - | - | 93 | 12 | H |
|  | 22 | + | + | + | + | - | + | + | + | - | - | - | + | - | - | 93 | 12 | A |
|  | 27 | + | + | + | + | - | + | + | + | - | - | - | + | - | - | 93 | 12 | A |
|  | 29 | + | + | + | + | - | + | + | + | - | - | - | + | - | - | 93 | 12 | A |
|  | 37 | + | + | + | + | - | + | + | + | - | - | - | (+/−) | - | - | 93 | 12 | A |
|  | 3 | + | + | + | + | - | + | + | + | - | - | - | + | - | - | 93 | 12 | A |
|  | 1 | + | + | + | + | - | + | + | + | - | - | - | + | - | - | 93 | 12 | A |
|  | 13 | + | + | + | + | - | + | + | + | - | - | - | + | - | - | 93 | 12 | A |
|  | 31 | + | + | + | + | - | + | + | + | - | - | - | + | - | - | 93 | 12 | A |
|  | 2a | + | + | + | + | - | + | + | + | - | - | - | + | - | - | 93 | 12 | M |
|  | 14 | + | + | + | + | - | + | + | + | - | - | - | + | - | - | 93 | 12 | L |
|  | 41 | + | + | + | + | - | + | + | + | - | - | - | + | - | - | 93 | 12 | L |
|  | 2b | + | + | + | + | - | + | + | + | - | - | - | + | - | - | 93 | 12 | M |
|  | 8 | + | + | + | + | - | + | + | + | -* | - | - | + | - | - | 93 | 12 | M |
|  | 18 | + | + | (+/−) | + | - | + | + | + | - | - | - | (+/−) | - | - | 93 | 12 | I |
|  | 12 | + | + | + | + | - | + | + | + | - | - | - | (+/−) | - | - | 93 | 12 | F + E |
|  | 25 | + | + | + | + | - | + | + | + | -* | - | -* | +* | - | - | 93 | 12 | E |
|  | 17 | + | + | + | + | - | + | + | + | -* | - | - | + | - | - | 93 | 12 | F |
| Laboratory ID number | 15 | + | + | + | + | - | + | + | + | - | - | - | + | - | - | 93 | 12 | G |
|  | 32b | + | + | + | + | - | + | + | + | - | - | - | + | - | - | 93 | 12 | G |
|  | 11 | + | + | + | + | - | + | + | + | - | - | - | + | - | - | 93 | 12 | B |
|  | 9 | + | + | + | + | - | + | + | + | - | - | - | + | - | - | 93 | 12 | D |
|  | 39 | + | + | + | + | (+/−) | + | + | + | - | - | - | + | - | - | 93 | 12 | D |
|  | 40 | + | + | + | + | - | + | + | + | - | - | - | + | - | - | 93 | 12 | D |
|  | 5 | + | + | + | + | - | + | + | + | - | - | - | + | - | - | 93 | 12 | D |
|  | 24 | + | + | + | + | - | + | + | + | - | - | - | (+/−) | - | - | 93 | 12 | C |
|  | 7a | + | - | - | + | - | + | + | + | - | - | - | - | - | - | 86 | 12 | J |
|  | 30 | + | - | - | + | - | + | + | + | - | - | - | - | - | - | 86 | 12 | J |
|  | 7c | (+/−) | - | - | + | - | + | + | + | - | - | - | - | - | - | 86 | 12 | I |
|  | 28 | + | + | - | + | - | + | + | + | - | - | + | - | - | - | 86 | 11 | B |
|  | 35 | - | - | - | (+/−) | - | + | + | (+/−) | - | - | - | - | - | - | 79 | 11 | A |
|  | 26 | + | + | + | + | - | + | + | + | + | - | - | + | - | - | 86 | 10 | F |
|  | 23 | + | + | + | + | - | + | + | + | +* | - | - | +* | - | - | 86 | 10 | F |
|  | 6a | + | + | + | + | + | + | + | + | -* | - | + | + | - | - | 86 | 10 | F |
|  | 6b | + | + | + | + | - | + | + | + | - | - | (+/−) | + | - | - | 86 | 10 | D |
|  | 45 | - | - | - | - | - | + | + | + | - | - | - | - | - | - | 71 | 10 | A |
|  | 36 | - | - | - | + | - | + | - | - | - | - | - | - | - | - | 64 | 9 | A |
|  | 43 | + | + | + | + | - | + | + | + | - | - | - | + | + | + | 79 | 8 | L |
|  | 4 | + | + | + | + | - | + | + | + | - | (+/−) | + | + | - | - | 79 | 8 | B |
|  | 16 | - | + | + | + | + | + | + | + | - | +* | +* | +* | - | - | 71 | 8 | F |
|  | 49 | + | + | + | + | - | + | + | + | - | - | (+/−) | + | + | + | 71 | 6 | K |
|  | % | 91.5 | 87.2 | 80.8 | 100 | 10.6 | 100 | 97.9 | 95.7 | 95.7 | 95.7 | 87.2 | 27.6 | 95.7 | 95.7 | Average | Average |  |
|  | Total | 43 | 41 | 38 | 47 | 5 | 47 | 46 | 45 | 45 | 45 | 41 | 13 | 45 | 45 | 89.34 | 11.49 |  |

α: SM162 preservant; β: Formulation-C preservant

(+/−): Equivocal result; Grey: False positive/negative result; −* : correct negative and positive for other flavivirus; +*: false positive and positive for other flavivirus

NEG: negative; DENV; dengue virus; TBEV: Tick borne encephalitis virus; YFV: yellow fever virus; JEV: Japanese encephalitis virus; WNV: West Nile virus

Assay code: A: PanBio indirect IgG ELISA; B: ELISA IgG indirect Dx Select Focus Diagnostic; C: DENV Detect ELISA InBios IgG; D: EIA Novatec indirect Dengue IgG; E: Euroimmun flavivirus IFA Mosaik; F: Euroimmune den 1–4 mosaik; G: Euroimmun anti DEN ELISA; H: Vircell IgG ELISA; I: ICT - SD Bioline Dengue Duo; J: Standard Diagnostics Dengue IgG ELISA; K: IBL IgG Germany; L: in house ELISA; M: in house IFA

Sample #14 is not used for score and % correct results calculation
